# Simulation‐Free Palliative Radiation Therapy: Implementing the Value‐Based and Easier‐Access Model of Care in a Rural Setting

**DOI:** 10.1002/jmrs.70011

**Published:** 2025-07-25

**Authors:** Matthew Fuller, Catherine Osbourne, Rachael Beldham‐Collins, Zoe Clarke, Yae Joo Jun, Denise Andree‐Evarts, George Warr, Wen‐Long Hsieh, Shiaw Juen Tan, Caitlin Allen, Rodney Hammond, Thomas Eade

**Affiliations:** ^1^ Central West Cancer Care Centre, Orange Health Service Orange New South Wales Australia; ^2^ Western Cancer Centre Dubbo New South Wales Australia; ^3^ The University of Sydney, Northern Clinical School, Sydney Medical School Sydney Australia; ^4^ Northern Sydney Cancer Centre, Royal North Shore Hospital St Leonards New South Wales Australia

**Keywords:** palliative radiation therapy, patient‐centre care, rural healthcare access, simulation‐free radiation therapy, value‐based healthcare

## Abstract

Palliative radiation therapy (RT) is a vital treatment modality for managing symptoms from metastatic disease, but access barriers and workflow inefficiencies can delay or preclude care delivery. Simulation‐free RT (SFRT) offers an effective, value‐based solution by eliminating the traditional computed tomography (CT) simulation process and utilising existing diagnostic or staging CT scans for treatment planning. This process reduces patient burden and accelerates time to treatment, prioritising patient‐centred care over traditional treatment pathways. Key technical considerations include managing dosimetric and geometric variations through appropriate patient selection and quality assurance processes. The successful implementation of SFRT requires a collaborative, multidisciplinary team approach, drawing on expertise from established centres to familiarise the team with the process. Access to diverse diagnostic imaging datasets and collaboration with various imaging providers is crucial. While careful patient selection is essential, our experience demonstrates that SFRT exemplifies value‐based healthcare principles by optimising resource utilisation while prioritising patient‐centred care, particularly valuable in rural settings where travel distances significantly impact treatment access. This paper aims to review the benefits and technical aspects, as well as provide key considerations for implementing SFRT in palliative RT settings.

## Graphical abstract

Palliative radiation therapy is a vital treatment modality for managing symptoms from metastatic disease, but access barriers and workflow inefficiencies can delay or preclude care delivery. Simulation‐free radiation therapy (SFRT) offers an effective, value‐based solution by eliminating the traditional simulation step and utilising existing diagnostic computed tomography (CT) scans for treatment planning. This paper aims to review the benefits and technical aspects, as well as provide key considerations for implementing SFRT in palliative radiation therapy settings.

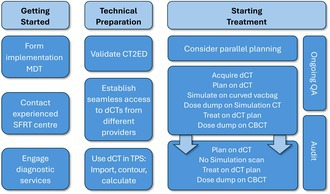

## 1 Background

Palliative radiation therapy (RT) is a proven, cost‐effective treatment that helps alleviate symptoms from bony and soft tissue metastases, such as pain, cord compression and uncontrolled bleeding [1, 2]. The delivery of RT is a complex process and requires a highly coordinated approach to provide a timely service and holistic patient care.

While solutions such as Rapid Access Clinics [3] and RT Advanced Practice [4] have streamlined certain aspects of RT, patients are still required to attend a conventional computed tomography (CT) simulation visit in person. Delays in this process may lead to compressed time for planning and quality assurance (QA) processes and hence increased pressure on multiple staff groups and/or postponed treatment start dates, potentially compromising the timeliness and efficacy of the palliative care.

Additionally, the extra visit can add considerable inconvenience for the patient/family/carer/system, resulting in increased medication requirements and the stress related to travel and accommodation requirements. The inconvenience of attending simulation and treatment can be so impactful that some patients may not be willing to undergo it, nor may be considered for this valuable treatment. In rural or regional areas, where patients are often distant from a radiotherapy centre, this issue worsens, leading to decreased RT utilisation with greater distance [5, 6, 7].

To address these challenges, simulation‐free RT (SFRT) has emerged as a valuable solution. While still considered a novel approach, it has been reported and validated widely in the literature [8, 9, 10, 11, 12, 13, 14, 15, 16, 17]. SFRT eliminates the need for a separate simulation step by using an existing diagnostic or staging CT scan (dCT) for dose calculations, enabling immediate treatment planning with less pressure on staff and a quicker path to treatment. It offers a value‐based approach by improving workflow efficiency and reducing patient burden while maximising clinical outcomes. This paper will delineate the key considerations for implementing SFRT and its associated benefits.

## 2 General Overview of SFRT

### 2.1 Benefits of SFRT

SFRT exemplifies the principles of value‐based healthcare (VBH), which, although defined in various ways [18, 19], consistently emphasises the efficient use of available resources to shift focus from outputs to outcomes. This approach prioritises what matters most to each individual and structures care around the person rather than around traditional treatment requirements. The core principles of VBH are evident in SFRT's ability to streamline processes and reduce the burden on patients, staff and the system, all while maintaining high‐quality care.

**FIGURE 1:**
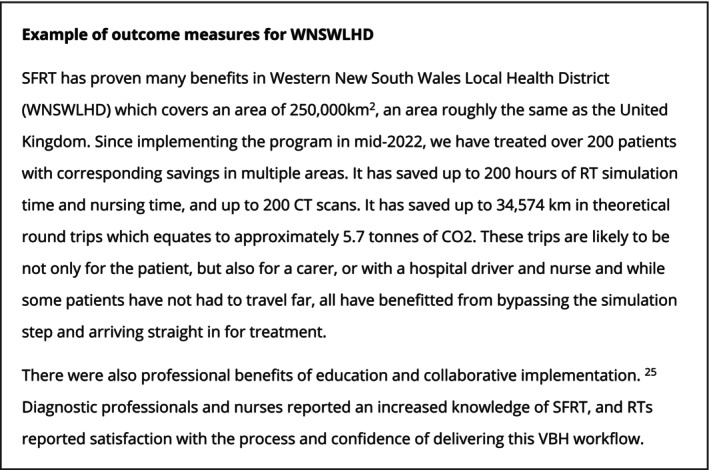
Examples of outcome measures in WNSWLHD.

For a range of potential benefits of SFRT, refer to Table 1. Figure 1 also contains some examples of outcome measures specifically for Western New South Wales Local Health District (WNSWLHD).

**TABLE 1:** Potential benefits of SFRT.

Benefits	Patient	Staff	Service	Environment
Quicker and easier access to RT	√		√	
Less pain and impact	√	√		
More time with family	√			
Less travel	√			√
Reduced accommodation needs and costs	√			
Reduced hospital stays	√		√	
Reduction in analgesia	√		√	
Less CT scans/radiation dose	√	√		√
Less staff stress in planning, QA		√	√	
Less staff time: RO, RT, Nursing		√	√	
Reduction in interhospital transport	√		√	√
Increased RT utilisation	√	√	√	
Opportunities for collaboration		√	√	

### 2.2 Understanding Variables in SFRT

To ensure the benefits of SFRT are achieved, understanding and addressing potential variables that can affect dose delivery is essential. Like any RT process, SFRT may experience discrepancies between the intended and actual delivered dose. These discrepancies can result from dose calculation inaccuracies, reproducibility issues, setup errors, motion and target delineation [20]. Understanding and factoring these variables into prescriptions, margins and daily verification imaging gives confidence in dose delivery.

In the context of SFRT, particularly, there are two primary types of variables to consider: dosimetric variations and geometric variations.

#### 2.2.1 Dosimetric Variations

Dosimetric variations occur when there are discrepancies in dose calculations due to factors such as differences in CT scanner image quality, particularly in CT number to electron density (CT2ED) relationships. While several authors conclude that minor dosimetric differences are often clinically acceptable and do not significantly affect treatment outcomes [10, 12, 14, 17], local measurements from a sample of scanners can help give confidence in the process.

Contrast media is often present in dCT scans but is typically absent at the time of treatment. To ensure accurate dose prediction in the absence of contrast, the areas containing contrast should be contoured, and an appropriate tissue‐equivalent density should be applied to these regions.

#### 2.2.2 Geometric Variations

**FIGURE 2:**
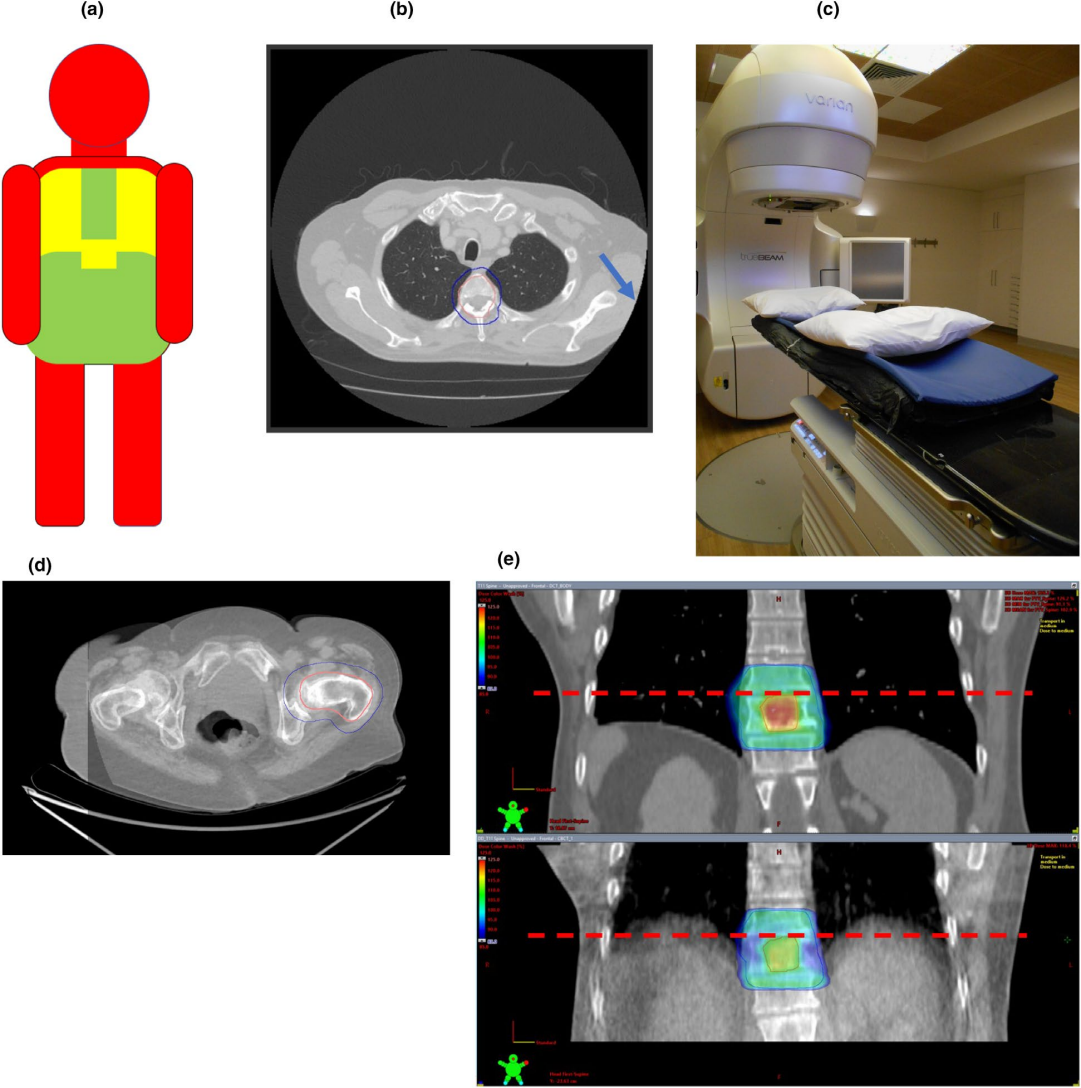
(a) Representation of the suitability of different treatment sites for SFRT. Areas shaded in green usually reproduce well from the dCT to treatment; areas in yellow are more variable, and so more thought is required, and areas in red are generally considered particularly variable and therefore unsuitable. (b) Adequate FOV for adequate visualisation and calculation. Note that some image deficit (indicated with a blue arrow) may be acceptable for SFRT planning, depending on the clinical situation—e.g., treatment angles could be chosen to avoid this area. (c) SFRT is set up with a curved full‐body vacuum bag. Using curved vacuum bags during treatment aims to more closely replicate the patient's planned dCT positioning on the treatment couch. (d) An example of a treatment verification CBCT: Utilising daily CBCT scans to verify and correct positioning gives confidence in the SFRT treatment delivery. Note the good agreement within and around the target regions in this example, despite discrepancies in less relevant areas. (e) An example of variation in diaphragm position on a T Spine plan. The dCT image (top) is in breath hold (note the clear delineation of the diaphragm in its stable position). The CBCT image (bottom) is free‐breathing (note the average position of the diaphragm) and results in a cooler distribution in this area.

Geometric variations occur when a patient's anatomy, including their external contour, does not align exactly with the dCT plan and are typically the most significant factors influencing treatment delivery. Although sometimes difficult to predict, many geometric variations can be mitigated to some extent through the use of appropriate solutions, such as:

- Clear eligibility criteria for SFRT (see Table 2)
  ○ Recent dCT available
  ○ Anatomical site appropriate (Figure 2a)
  ○ Adequate field of view (FOV) on dCT (Figure 2b)
- Curved vacuum bags for treatment, to replicate CT couch‐tops used in dCT (Figure 2c)
- Daily cone beam CT (CBCT) verification (Figure 2d)
- Six degrees of freedom correction is ideal
- Considerations of lung and diaphragm when dCT is in breath hold (Figure 2e)
- Detailed setup instructions
- Maintain adequate planning target volume (PTV) margins

**TABLE 2:** WNSWLHD SFRT inclusion and exclusion criteria (Adapted from Wong et al. [9]).

Criteria	Considerations	Inclusion criteria	Exclusion criteria
Performance status	Patient's general condition and other needs (e.g., pain relief and travel)	ECOG status ≤ 3	ECOG status > 3
Scan recency	Clinically reliable for patient and disease anatomy Older dCT datasets may be used with clinical reasoning (e.g., older dCT used together with recent MRI for disease progression) Consider requesting an updated dCT scan at a location close to patient's home	Diagnostic CT, PET/CT, or SPECT/CT scan ≤ 2 months	Diagnostic CT, PET/CT, or SPECT/CT scan is > 2 months from treatment date (at discretion of RO)
Treatment site reproducibility	Crucial for patient selection [8, 9] Patient positioning Motion and variability in patient position (consider general image fusion experience) Consider breath hold and diaphragm location	Bony and fixed soft tissue sites below and inclusive of T4, which do not require a thermoplastic mask or vac‐bag for stabilisation. These sites may include: Abdomen – Chest – Groin – Hip – L Spine – Pelvis – Sacrum – T Spine	The patient's position on the dCT image is deemed non‐reproducible for treatment Painful bony and soft tissue sites that require the use of a thermoplastic mask and extensive stabilisation equipment: – Brain – C Spine – Lungs – Upper and Lower Extremities
Dataset adequate	Can the treatment planning system calculate on the dataset? Is the external contour largely visible, or too limiting?	Calculation possible Adequate visualisation of the area to be treated Adequate coverage in the sup‐inf direction Adequate coverage in the Field of View (i.e., is the external contour adequate in the treatment area for beam entry?)	Calculations not possible The treatment site was not adequately visualised
Dose/fractionation	Palliative dose/fractionation, with/without an integrated boost to GTV	8Gy in 1 fraction 20Gy in 5 fractions 20Gy in 4 fractions	> 5 fractions

### 2.3 Techniques for SFRT

A key feature of SFRT is its adaptability to existing treatment protocols. Centres can seamlessly integrate SFRT into their current workflows without altering standard treatment techniques. For example, departments that use single postero‐anterior (PA) fields for spine treatments can continue employing this method within the SFRT framework.

At WNSWLHD, we have found that volumetric modulated arc therapy (VMAT) has proven particularly effective in managing variations in external contour and positional changes. This technique ensures the delivery of a uniform dose across target volumes, often incorporating an integrated gross tumour volume (GTV) boost while sparing organs at risk (OARs) such as the bowel and spinal cord. This approach is crucial in reducing acute toxicity, ensuring a high‐quality plan and optimising clinical outcomes.

### 2.4 Fractionation in SFRT

SFRT is most efficient when delivered as a single fraction. The removal of the simulation step expedites the planning process, and the relative time savings diminish as the number of fractions increases. Single fractions, especially for bone metastases, are often underutilised [21, 22]. While Chand et al. [7] argue that a single fraction may represent a discrimination of patients who live far away from RT centres, delivering a low‐impact single SFRT fraction can offer a patient‐centred and value‐based solution in most situations. For patients who experience good relief from the single fraction, re‐treatment with further low‐impact SFRT fractions is feasible.

### 2.5 Verification of SFRT

Like for conventionally simulated treatment, RTs are well‐equipped and uniquely positioned to make informed holistic treatment decisions for SFRT by utilising their expertise in patient care, dosimetry and three‐dimensional imaging data. Daily CBCT plays a critical role in verifying target and PTV alignment, allowing RTs to make necessary setup adjustments to ensure accurate treatment delivery. In our experience, the need to return to simulation is rare, as the SFRT process is designed to minimise this. However, maintaining the option to return to simulation can be useful in addressing any significant anatomical changes that might arise. This approach ensures that treatment remains accurate, optimising clinical outcomes while maximising resource utilisation.

### 2.6 Limitations of SFRT

SFRT offers a valuable approach to palliative care, particularly in rural settings. However, several limitations should be considered:

Achieving precise patient positioning without traditional simulation can be challenging. While adequate margins and careful patient selection can mitigate this issue to some extent (see Table 2 and Figure 2a), SFRT treatments may take longer than a conventionally simulated patient [8, 17].

Additionally, the nature of palliative RT means that some patients pass away soon after treatment. Although this may suggest potential issues with patient selection [23], it is important to note that a significant proportion of these patients experience pain relief [24]. SFRT therefore offers patient‐centred care by simplifying access to treatment, reducing the burden of extra visits and potentially improving the patient's last days by minimising travel and discomfort.

## 3 Implementation Considerations

**FIGURE 3:**
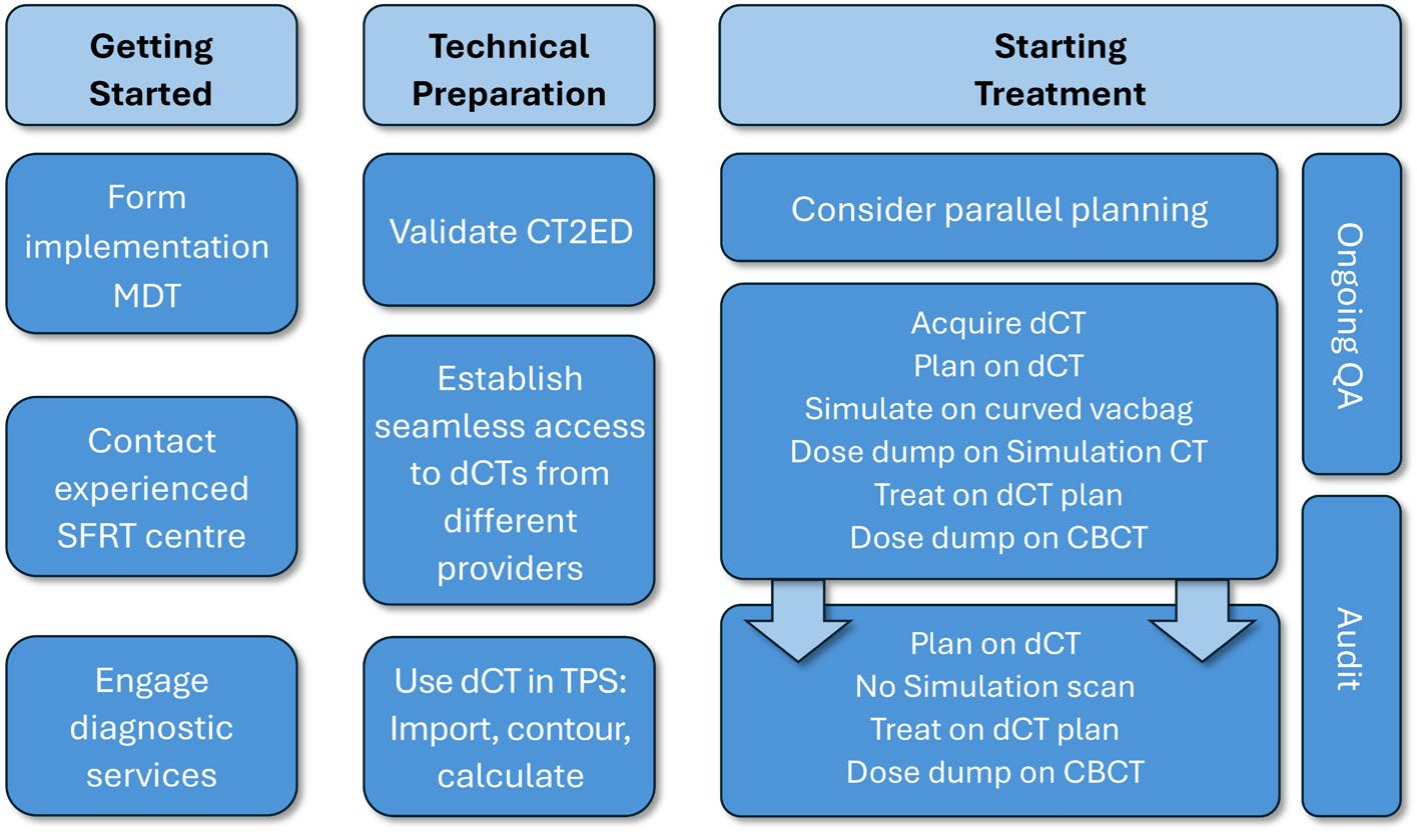
SFRT implementation process. Note that the steps are not necessarily sequential and are sometimes fluid. CBCT, cone beam CT; CT, computed tomography; CT2ED, CT number to electron density relationship; MDT, multidisciplinary team; dCT, diagnostic or staging CT scan; SFRT, simulation‐free radiation therapy; TPS, treatment planning system.

While SFRT is an established and validated process, confidence in its implementation requires careful planning and collaboration across various teams and departments. Below are some key considerations for a successful implementation of SFRT into practice. See Figure 3 for a summary of the main steps.

### 3.1 Forming the Service Development Team

A multidisciplinary team (MDT) approach is crucial for the successful implementation and understanding of the SFRT process. Strong leadership, clear communication and enthusiasm from all team members, including radiation oncologists (ROs), radiation oncology medical physicists (ROMPs) and radiation therapists (RTs), are essential. Collaboration among these team members ensures that the SFRT process is well understood and successfully implemented.

ROs are pivotal to patient and image selection, ROMPs ensure the dosimetric validity of treatment and RTs are heavily involved in establishing the entire SFRT pathway—right from booking the patient and acquiring and evaluating datasets to planning and delivering the treatment.

### 3.2 Engage With Experienced SFRT Centres

Published literature is helpful in guiding the development of any new technique; however, it is also beneficial to draw from the first‐hand experience of centres already familiar with the process. This could include site visits to learn from other MDTs who are experienced in SFRT, or virtual meetings which are also very effective, particularly for rural or interstate connections.

Establishing and maintaining these relationships with different centres and with different individuals is valuable not only for the initial implementation process but also for ongoing collaboration.

### 3.3 Collaboration With Diagnostic Departments

Similarly, it is a great opportunity for collaboration and learning with our diagnostic colleagues [25]. Relationships can be established in face‐to‐face and virtual education sessions. These professional relationships may then translate into having quick access to the different imaging services when a problem may arise (e.g., to ask for a reconstruction with a different FOV, or a different dataset to be sent).

### 3.4 Technical Image Access

Gaining electronic access from various providers in a compatible format for the treatment planning system (TPS) is essential. As there are many different potential providers and variations between private and public access, this access takes time to establish. A strong relationship with local Information Technology (IT) is useful to ensure a smooth workflow.

Different imaging modalities like CT, PET‐CT and SPECT, may be used, each requiring specific handling to integrate into the TPS. Additionally, technical issues such as CT gantry tilt or image straightening may need to be addressed to allow treatment planning.

### 3.5 Validating the SFRT Process

**FIGURE 4:**
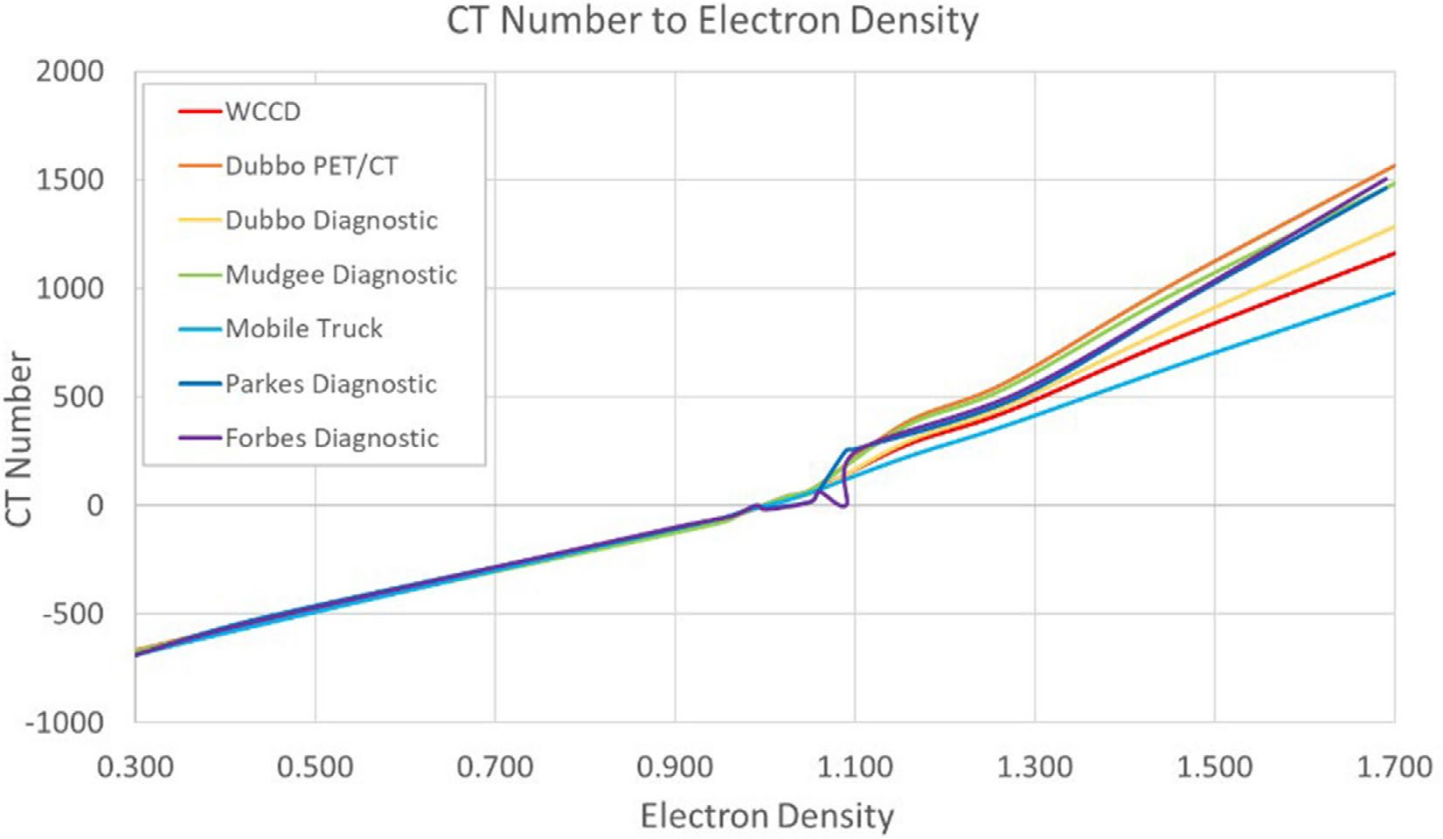
Examples of CT to electron density curves measured for various scanners in WNSWLHD.

While the reliability of SFRT dose calculations has been demonstrated, it is important to conduct local validation. In WNSWLHD, small multidisciplinary teams visited different private and public diagnostic departments and performed CT2ED measurements. It was found that the curves largely coincided, and differences were limited to the very high densities (see Figure 4). Any subsequent dosimetric differences were thought to be clinically acceptable for this cohort of palliative patients, confirming findings in the SFRT literature [10, 12, 14, 17].

**FIGURE 5:**
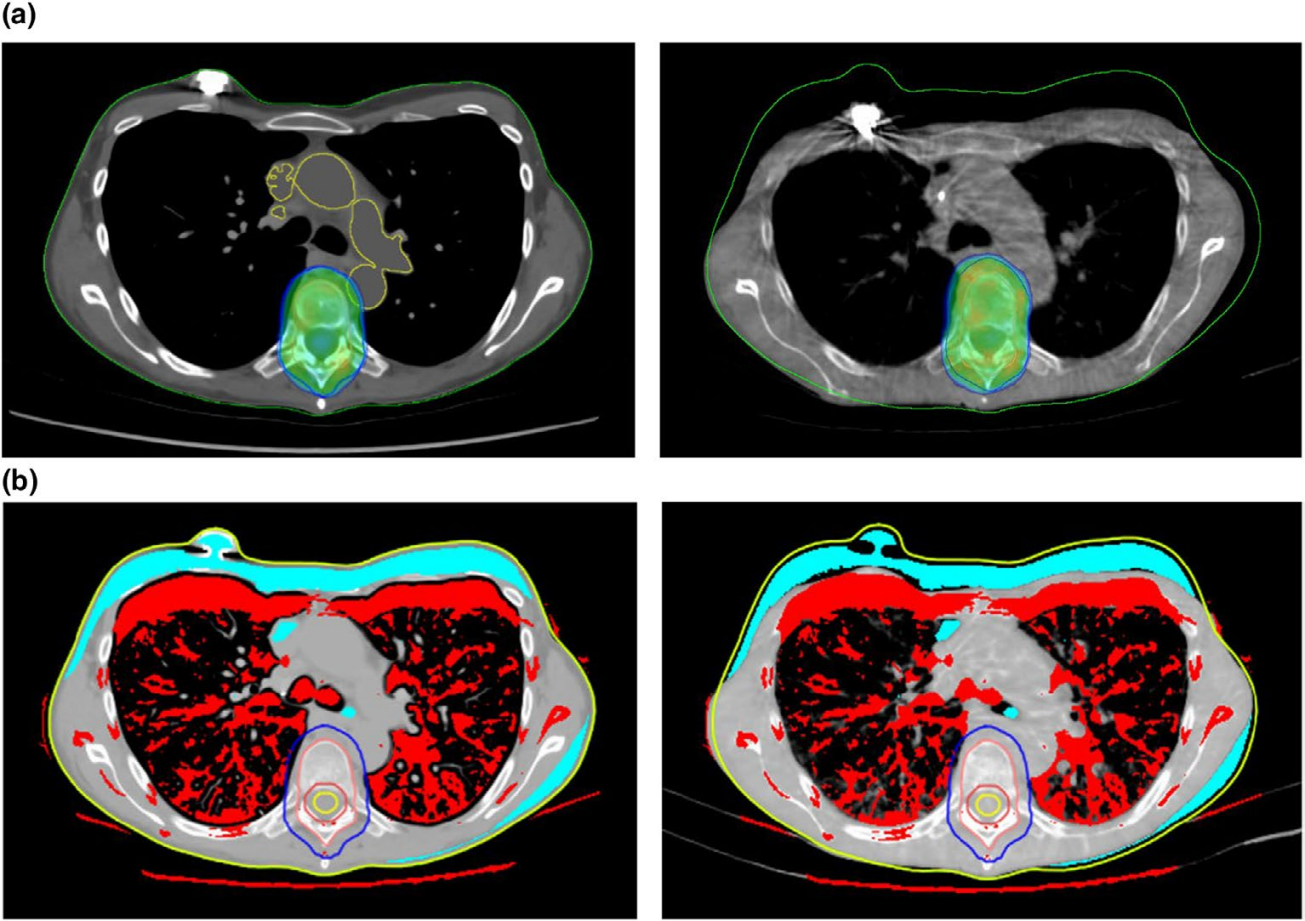
(a) An example of colour wash dose distributions on dCT (left) and dose dump on CBCT (right). The dose range displayed is 90%–110% of the target dose, in this case 8Gy. Contrast has been contoured and forced to a soft tissue density in the dCT plan. Note the differences in external contour and other anatomy on the CBCT plan, but the relatively similar dose distributions. (b) An example of Mobius CT data matches with the dCT (left) and CBCT (right). The external and target contours are both from the original dCT plan. Note the representation of differences in CT values.

Also, as part of establishing each step of the process, a period of parallel planning similar to Wong et al. [9] can be helpful. As the booking for palliative RT is received, the existing dCT dataset is retrieved from the imaging provider and evaluated. If it meets the inclusion criteria (see Table 2), a plan including contours can be generated on the dCT as the planning CT. The patient can be simulated in the diagnostic position with a curved vacuum bag, and the plans compared. A final QA step is to copy the plan onto the day 1 treatment verification CBCT (dose dump) to compare with the dCT and planning CT plans (see Figure 5a) [26, 27, 28]. A range of treatment sites and patient types is appropriate for this process, with the aim of removing the simulation step.

### 3.6 Ongoing QA

Once confident with the dosimetric impact of the various providers and the simulation step is removed, regular QA is essential to ensure treatment accuracy and reassure staff, especially when geometric variations arise. The accuracy of the CBCT dose dump process should be evaluated locally [29], and it is also a valuable ongoing quality assurance (QA) process after removing the simulation step for all or select cases (see Figure 5a).

Another option is the use of automatic dose calculation tools such as Varian Mobius. This efficient QA tool assesses the agreement of the CT datasets (see Figure 5b) as well as the mean dose delivered to the target [30]. Both the dose dump and Mobius calculations have inherent limitations (e.g., limited CBCT FOV) but are important in maintaining QA. Various metrics should be developed for these QA plans, which can help identify discrepancies and prompt further investigation. Routine audits and continuous QA processes are also essential for maintaining the integrity of SFRT delivery.

## 4 Conclusion

In conclusion, SFRT offers a transformative approach to palliative RT, particularly in rural and underserved areas. By reducing the barriers to treatment, such as unnecessary in‐person simulation visits, SFRT improves patient access to care and enhances the efficiency of the planning and treatment process. This approach aligns well with value‐based healthcare principles, ensuring that patients receive timely, high‐quality care with minimal disruption to their lives, making it a valuable model for the future of RT.

## Conflicts of Interest

The authors declare no conflicts of interest.

## Data Availability

Data sharing not applicable to this article, as no datasets were generated or analysed during the current study.
